# Characterization and Hydration Mechanism of Ammonia Soda Residue and Portland Cement Composite Cementitious Material

**DOI:** 10.3390/ma14174794

**Published:** 2021-08-24

**Authors:** Dong Xu, Pingfeng Fu, Wen Ni, Qunhui Wang, Keqing Li

**Affiliations:** 1 School of Energy and Environmental Engineering, University of Science and Technology Beijing, Beijing 100083, China; sdlycsxd@163.com (D.X.); wangqh59@163.com (Q.W.); 2 Beijing Key Laboratory on Resource-Oriented Treatment of Industrial Pollutants, University of Science and Technology Beijing, Beijing 100083, China; 3 Key Laboratory of the Ministry of Education of China for High-Efficient Mining and Safety of Metal Mines, University of Science and Technology Beijing, Beijing 100083, China; pffu@ces.ustb.edu.cn (P.F.); Lkqing2003@163.com (K.L.); 4 School of Civil and Resource Engineering, University of Science and Technology Beijing, Beijing 100083, China

**Keywords:** ammonia soda residue, hydration mechanism, cementitious material, resource utilization, CO_2_ emissions

## Abstract

The use of ammonia soda residue (ASR) to prepare building materials is an effective way to dispose of ASR on a large scale, but this process suffers from a lack of data and theoretical basis. In this paper, a composite cementitious material was prepared using ASR and cement, and the hydration mechanism of cementitious materials with 5%, 10%, and 20% ASR was studied. The XRD and SEM results showed that the main hydration products of ASR-cement composite cementitious materials were an amorphous C-S-H gel, hexagonal plate-like Ca(OH)_2_ (CH), and regular hexagonal plate-like Friedel’s salt (FS). The addition of ASR increased the heat of hydration of the cementitious material, which increased upon increasing the ASR content. The addition of ASR also reduced the cumulative pore volume of the hardened paste, which displayed the optimal pore structure when the ASR content was 5%. In addition, ASR shortened the setting time compared with the cement group, and the final setting times of the pastes with 5%, 10%, and 20% ASR were 30 min, 45 min, and 70 min shorter, respectively. When the ASR content did not exceed 10%, the 3-day compressive strength of the mortar was significantly improved, but the 28-day compressive strength was worse. Finally, the hydration mechanism and potential applications of the cementitious material are discussed. The results of this paper promote the use of ASR in building materials to reduce CO_2_ emissions in the cement industry.

## 1. Introduction

Ammonia soda residue (ASR) is a solid waste produced during the preparation of soda ash by the ammonia-soda method. Its main components are CaCO_3_, CaSO_4_, NaCl, and CaCl_2_, and its pH value is generally in the range of 10–12 [1]. In China, the annual growth rate of ASR exceeds 5 million tons, and the accumulated amount exceeds 50 million tons due to the lack of effective treatment methods (the current treatment efficiency is only 3%–4%) [2]. The accumulation of such a large amount of ASR poses a potential environmental pollution hazard due to the storage area required, and the impact on groundwater and air increases each day [3]. Because the production of soda ash by the ammonia method requires large amounts of chloride salts as raw materials, most soda plants are located near the sea to conveniently obtain chloride salts; therefore, ASR is piled on beaches, which threatens the ocean environment and also poses hidden dangers in the form of dam breaches [4]. Thus, the treatment and utilization of ASR have become a popular research topic.

Many studies have investigated the treatment and utilization of ASR, such as extracting calcium components [5], and using it to prepare desulfurizers [6] and soil regulators (pH regulators, heavy metal solidification) [7,8], but these methods produce secondary pollution or face small-scale issues. Some researchers have studied the use of ASR to prepare building materials. Zhao [9] prepared a geopolymer from ASR and fly ash that displayed good volume stability and found that ASR improved the comprehensive performance of the geopolymer, and when the mass ratio of ASR to fly ash was 1:4, the 60-day and 180-day compressive strength reached 13.5 MPa and 18 MPa, respectively. Wang [10] studied the application of ASR for the preparation of cement and prepared cement that satisfied the requirements of 52.5-grade cement; however, the impermeability and freeze–thaw resistance of the cement were poor. Guo [11] utilized soda solid waste ASR and calcium carbide slag to synergistically activate ground granulated blast-furnace slag (GGBS) and fly ash (FA). The 28-day compressive strength of the binder system reached 17.5–43.2 MPa. Xu [12] used ASR, GGBS, steel slag, and flue gas desulfurization gypsum as cementitious materials and waste iron ore tailings as an aggregate to prepare clinker-free concretes. The compressive strength of the optimized concrete reached 36.29 MPa and 66.31 MPa after 3 and 360 days, which were, respectively, 32% and 27% higher than the control specimen. 

From the perspective of the treatment scale, the use of ASR to prepare building materials is the only way to effectively reuse ASR. At present, cement is the main artificial building material throughout the world, but its production consumes natural resources and produces CO_2_ emissions. In fact, the cement industry has always been one of the main sources of CO_2_ emissions [13]. According to statistics, each ton of produced cement emits 900 kg of CO_2_, and the total amount of CO_2_ emissions from cement production accounts for more than 5% of the world’s total annual CO_2_ emissions [14]. Methods to reduce the production of cement is a current and future research hotspot, so it is particularly important to select appropriate materials to completely or partially replace cement to prepare building materials, such as using supplementary cementitious materials (SCMs) (e.g., GGBS, SS, and FA) [15]. In this regard, the use of solid waste to replace cement to prepare building materials can reduce the amount of cement, thereby reducing CO_2_ emissions associated with cement production processes. Therefore, the use of ASR to replace part of cement to prepare building materials can reuse ASR on a large scale and also reduce the amount of cement, which is highly feasible in terms of technology, costs, and policy. In particular, two policies are taken into account: (1) environmental protection tax (25 yuan per ton of solid waste) in China since 2018; (2) the Chinese government has made a solemn commitment to strive for the peak of carbon dioxide emissions by 2030 and to achieve carbon neutrality by 2060.

Despite these goals, there are some deficiencies in the current research on the preparation of building materials from ASR, such as the high costs of cement burning, or the complex preparation process; therefore, a simple inexpensive treatment process is needed to solve the current problem of ASR stacking. The purpose of this paper was to provide theoretical data support for the preparation of cementitious materials from ASR via a simple and inexpensive method to reuse ASR and reduce CO_2_ emissions of the cement industry. To this end, an ASR-cement composite cementitious material was prepared. The hydration mechanism and hydration characteristics of the composite cementitious material were analyzed by XRD, SEM, TG-DSC, and MIP. The physical properties of the mortar prepared by the ASR-cement composite cementitious material were studied. Finally, the potential applications of the ASR-cement cementitious material were discussed.

## 2. Raw Materials and Methods

### 2.1. Raw Materials

The cement used in this work was P.O 42.5 cement produced by the China United Cement Group Co., Ltd. (Beijing, China), with a Blaine fineness of 351 m^2^/kg. The ASR was obtained from Tangshan Sanyou Chemical Co., Ltd. (Tangshan, China), which is one of the largest soda ash manufacturers in China. The ASR had a moisture content of 25%–50%, dried and ground to a powder with a Blaine fineness of 370 m^2^/kg. The chemical compositions of ASR and cement are shown in Table 1. The main chemical constituents of ASR are CaO, MgO, and SiO_2_, with a chloride content of 11.5%. 

**Figure 1:**
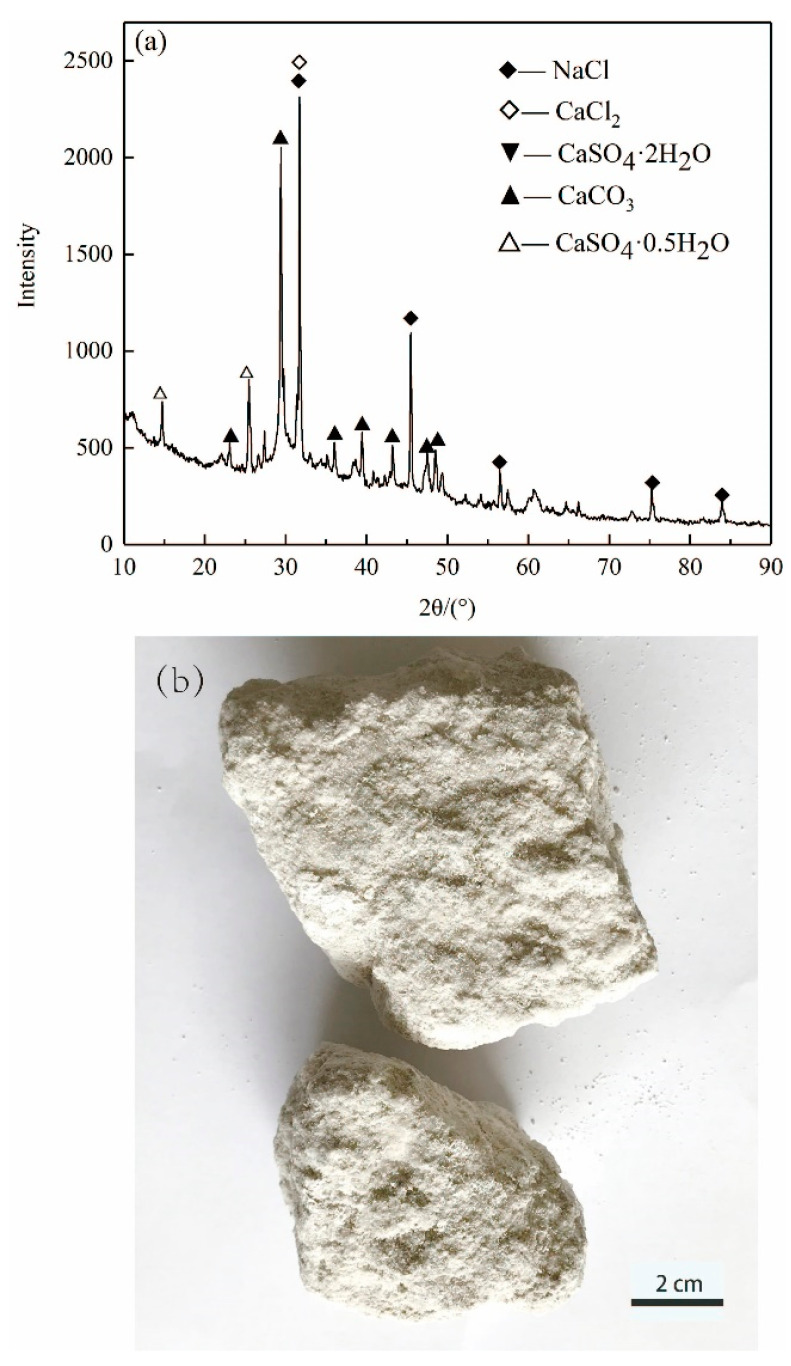
(**a**) XRD pattern and (**b**) photo of ASR.

Figure 1 shows the morphology and XRD pattern of ASR, which is gray-white in appearance. The mineral phases are mainly CaCO_3_, NaCl, CaSO_4_·2H_2_O and CaSO_4_·0.5H_2_O. The aggregate used in this study was an ISO reference sand with a particle size smaller than 2 mm according to ISO R679 standard. It was purchased from Xiamen ISO Standard Sand Co., Ltd. (Xiamen, China).

### 2.2. Mix Proportions and Specimen Preparation

After the ASR was crushed and dried, it was ground to a specific surface area of 370 m^2^/kg using a grinder mill. The ASR and cement were weighed according to the cementitious material ratio in Table 2 and then mixed with water. The water-binder (*w/b*) ratio was 0.5. The mixture was put into a planetary mixer (NJ-160) for cement paste and mixed at a low speed for 120 s, stopped for 15 s, and then mixed at a high speed for 120 s. The paste sample was poured into a 30 mm × 30 mm × 50 mm iron mold for trial molding and then transferred to a constant-temperature and humidity curing box for storage. The curing temperature was 20 ± 2 °C, and the relative humidity was ≥90%. At 3 d and 28 d, the paste samples were taken out and then crushed and put into anhydrous ethanol to terminate the hydration reaction and characterize the hydration mechanism. Cementitious material (450 g) was weighed according to the ASR-and-cement ratios in Table 2. Then, 1350 g standard sand and 225 g water were added, and a planetary mixer (JJ-5) was used to mix the mortars evenly. The mixing time was 30 s at low-speed, high-speed stirring for 30 s, pausing for 90 s, and high-speed stirring for 60 s. After the mortar was prepared, it was immediately poured into an iron mold with a size of 40 mm ×40 mm ×160 mm. After curing for 1 d at 20 ± 2 °C and a relative humidity of ≥90%, the mortar was demolded and then placed in 20 ± 1 °C water to cure for 3 d, 7 d, and 28 d for compressive strength testing. The setting time of the cementitious material was tested in accordance with the Chinese standard GB/T 1346-2011, and the compressive strength tests were carried out in accordance with GB/T 17671-2020.

### 2.3. Testing Methods

The hydration mechanism of the composite cementitious materials was characterized by XRD, SEM, TG-DSC, and MIP. The mineral phases of materials were characterized by an Ultima IV X-ray diffractometer equipped with a copper target working at 40 kV and 40 mA. Scanning was carried out for 2θ values from 10° to 90° at a scanning rate of 10° min^−1^. The microstructure of pastes was analyzed by a SUPRA55 field emission scanning electron microscope. The sample preparation method for SEM is as follows: the paste sample was crushed into thin slices with a diameter of about 1–2 mm and a thickness of 0.3–0.5 mm. Then, the sample was placed in a drying oven and dried to a constant weight at a constant temperature of 65 °C. Finally, conductive glue was used to fix the sample to the sample stage, and the surface of the sample was evenly sputtered with gold to render it conductive. TG-DSC analysis was carried out by an STA409C/CD differential scanning calorimeter, and the testing temperature was increased from 20 °C to 1000 °C at a rate of 10 °C/min under an inert nitrogen (N_2_) atmosphere. The pore structures of hardened pastes were measured by a Quantachrome Autoscan-33 mercury intrusion porosimeter. The heat of hydration of composite cementitious materials was measured by a TAM-Air isothermal calorimeter at a constant temperature of 25 °C with a 0.5 w/b ratio according to the ASTM C1702 standard test method.

## 3. Results

### 3.1. XRD Analysis

**Figure 2:**
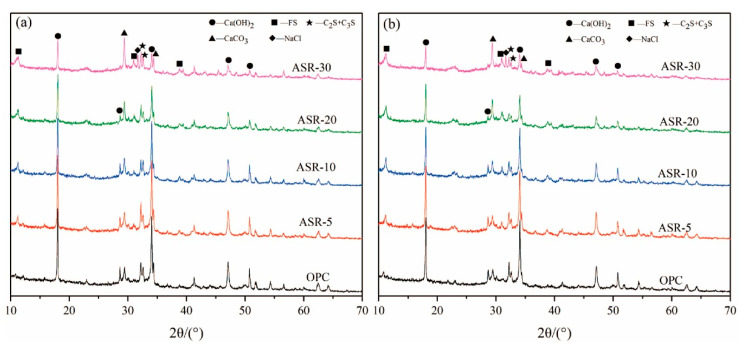
XRD patterns of ASR-cement composite cementitious materials: (**a**) 3 days; (**b**) 28 days.

The XRD patterns of the paste samples with 0, 5%, 10%, 20%, and 30% of ASR contents at 3 days and 28 days are shown in Figure 2. The results show that the hydration products of OPC are mainly C-S-H gel and Ca(OH)_2_ (CH). The C-S-H gel has no characteristic diffraction peaks in the XRD pattern because it is amorphous. A new hydration product, Friedel’s salt (FS), appeared in the ASR-cement composite cementitious material control OPC. According to the classification of substances with typical characteristic XRD peaks, there are mainly three types of substances: (1) Hydration products—mainly dicalcium silicate and tricalcium silicate, which are hydrated to form CH, and the new hydration product FS; (2) The active mineral components that were not hydrated—C_3_S and C_2_S; (3) Materials that do not possess pozzolanic activity and cannot be hydrated—CaCO_3_ and NaCl.

From Figure 2a, after curing for 3 days, the amount of CH changed significantly upon increasing the amount of ASR, especially when the ASR content exceeded 10% (ASR-10). The intensity of the CH characteristic diffraction peaks gradually decreased, indicating that the CH content in the hardened paste gradually decreased. The intensity of the CaCO_3_ diffraction peak gradually became stronger upon increasing the ASR content due to overlap of the CaCO_3_ diffraction peak in the ASR and the CaCO_3_ diffraction peak generated from carbonization during curing. Similarly, the characteristic diffraction peak of NaCl also gradually increased upon increasing the ASR content, indicating that a large amount of NaCl remained without participating in the hydration reaction. In Figure 2b, after curing for 28 days, the characteristic diffraction peaks of various minerals showed roughly the same trend as those in the sample cured for 3 days, indicating that the hydration products of ASR-cement composite cementitious materials do not change significantly during the later stage of hydration.

### 3.2. SEM Analysis

**Figure 3:**
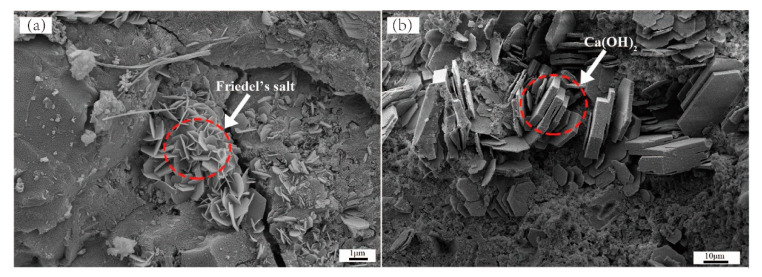
SEM image of hardened paste of ASR-10 cementitious material, the morphology of Friedel’s salt (**a**); and Ca(OH)_2_ (**b**).

The XRD results showed that the early and late hydration products of the ASR-cement composite were basically the same, and the types of hydration products did not change with the ASR content; therefore, the samples with an ASR content of 10% (ASR-10) and a hydration age of 3 days were selected for hydration product morphology observations. Figure 3 shows SEM images of the ASR-10 hardened paste of the ASR-cement composite. In Figure 3a there are many regular hexagonal plate-shaped FS particles with a diameter of about 1–2 μm, while there are also hexagonal plate-shaped substances in Figure 3b; however, they have a coarser appearance with a diameter of 5–10 μm, which is a typical CH crystal morphology. Around the CH crystals, there is an amorphous, honeycomb-like substance, which was the typical morphology of a C-S-H gel in the early stage. The formation of CH and FS has an important effect on the density of the paste, because they will fill the pores of the C-S-H gel with unreacted particles and form a dense structure, which plays an important role in the strength development of the paste.

### 3.3. TG-DSC Analysis

**Figure 4:**
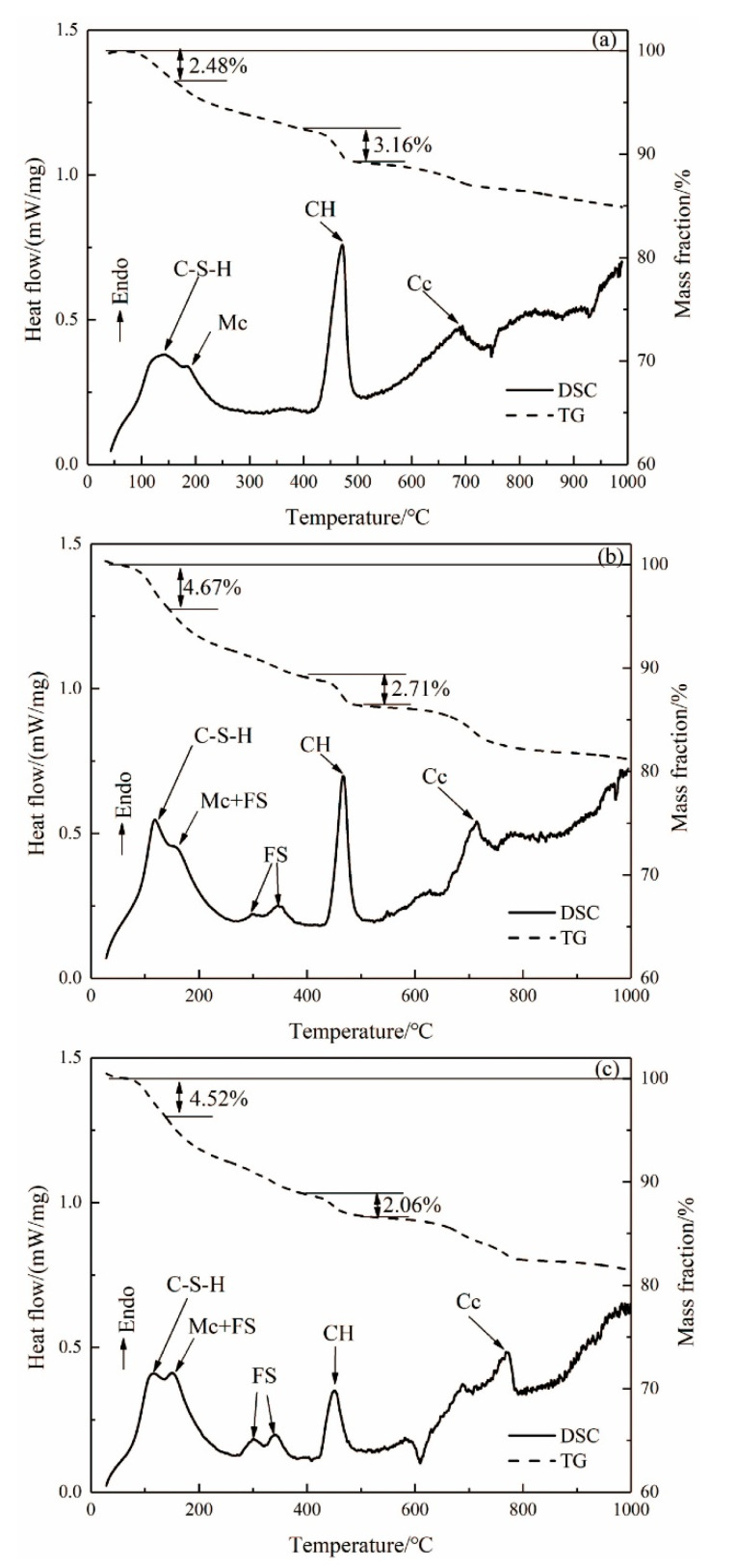
DSC and TG curves of (**a**) OPC paste, (**b**) ASR-10 paste, and (**c**) ASR-20 paste (Mc: monocarbonate; FS: Friedel’s salt; CH: Ca(OH)_2_; Cc: CaCO_3_).

The TG-DSC curves of the hardened pastes of ASR-cement composite cementitious material after aging for 3 days are shown in Figure 4. From the DSC curve in Figure 4a, the exothermic peak near 120 °C represents the dehydration of the C-S-H gel. The endothermic peak near 200 °C represents the decomposition of monocarbonate (Mc), and the exothermic peak near 450 °C is caused by the decomposition of CH, while the exothermic peak above 600 °C is caused by the decomposition of CaCO_3_. It can be seen from Figure 4b,c that the endothermic decomposition peak of FS appeared in the DSC curves of ASR-10 and ASR-20 from 230–410 °C, which is consistent with the XRD and SEM results. The CaCO_3_ in OPC samples were generated by carbonization of the hardened paste during curing, while the CaCO_3_ in ASR-10 and ASR-20 samples were not only carbonized but also introduced from ASR.

It can be seen from the TG curves in Figure 4 that the weight loss rates of OPC, ASR-10, and ASR-20 samples in the temperature range of 25–150 °C were 2.48%, 4.67%, and 4.52%, respectively; however, the results do not mean that the addition of ASR increased the amount of C-S-H gel in the pastes, because the CaSO_4_·2H_2_O in ASR will also be dehydrated in this temperature range. The total weight loss rates of the three groups of samples in the temperature range of 25–1000 °C were 15.11%, 18.79%, and 18.68%, respectively. These do not indicate the number of hydration products in the samples, because the amounts of C-S-H gel, CH, FS, and CaCO_3_ are different, and the weight loss of each substance during decomposition is also different. 

In cement-based materials, the CH quantitative analysis method is often used to characterize the degree of hydration of cementitious materials. The weight loss in the temperature range of 400–500 °C is caused by the dehydration of CH, so the weight loss within this range can be used to calculate the CH content of the paste [16]. The FS content in the paste can be determined using the weight loss value in the interval of 230–410 °C, because FS generally loses six interlayer water molecules in this interval [17]. The amount of CH ($P_{\mathrm{Ca}{\left(\mathrm{OH}\right)}_{2}}$, in wt.%) formed in the paste can be calculated from the DSC curves using Equation (1) [18], and the Friedel’s salt content formed in the paste can be calculated from the DSC curves using Equation (2) [19]:

(1) $$P_{\mathrm{Ca}{\left(\mathrm{OH}\right)}_{2}}{=WL}_{\mathrm{Ca}{\left(\mathrm{OH}\right)}_{2}}\frac{{\mathrm{MW}}_{\mathrm{Ca}{\left(\mathrm{OH}\right)}_{2}}}{{\mathrm{MW}}_{H_{2}O}}$$

where ${WL}_{\mathrm{Ca}{\left(\mathrm{OH}\right)}_{2}}$ is the mass loss from the hydroxylation of Ca(OH)_2_ (wt.%), and ${\mathrm{MW}}_{H_{2}O}$ and ${\mathrm{MW}}_{\mathrm{Ca}{\left(\mathrm{OH}\right)}_{2}}$ are the molar masses of H_2_O and Ca(OH)_2_, respectively.

(2) $$M_{\mathrm{FS}}=\frac{M_{\mathrm{FS}}}{{6M}_{H_{2}O}}m_{H_{2}O}$$

where $m_{H_{2}O}$ is the mass loss from the DSC curves of the main-layer water in Friedel’s salt (wt.%), and $M_{\mathrm{FS}}$ and $m_{H_{2}O}$ are the molar masses of Friedel’s salt and water, with values of 561.3 g/mol and 18 g/mol, respectively.

**Figure 5:**
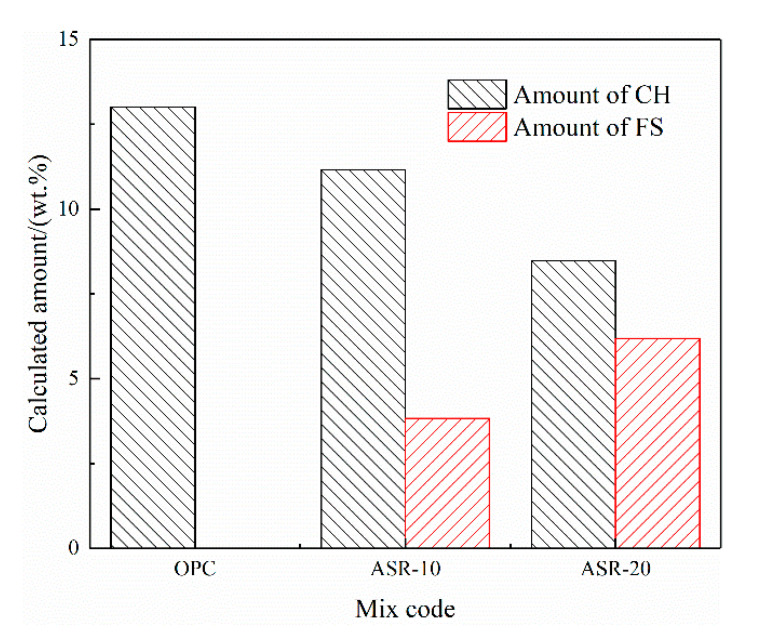
Calculated amounts of Ca(OH)_2_ and Friedel’s salt in the ASR-cement hardened pastes.

Figure 5 shows the CH and FS contents of the hardened paste of ASR-cement composite cementitious material calculated by Equations (1) and (2). Assuming that ASR acts as an inert material and does not participate in the hydration reaction, in theory, the CH content in ASR-10 and ASR-20 should be 90% and 80% of the OPC, respectively; however, the amounts of CH in ASR-10 and ASR-20 were 85.7% and 65.15% of the OPC, respectively, which are lower than the theoretical values. This indicates that the addition of ASR consumed CH in the paste, but it does not mean that the hydration degree of cement was reduced. Similarly, the theoretical FS content of the ASR-20 group should be twice that of the ASR-10 group, but the FS contents of ASR-10 and ASR-20 pastes in Figure 5 were 3.83% and 6.17%, respectively. The latter value is much lower than the theoretical value, which is due to the amount of aluminate (C_3_A) in cement, because the formation of FS is related to the aluminate-to-chloride ratio.

### 3.4. Hydration Heat

**Figure 6:**
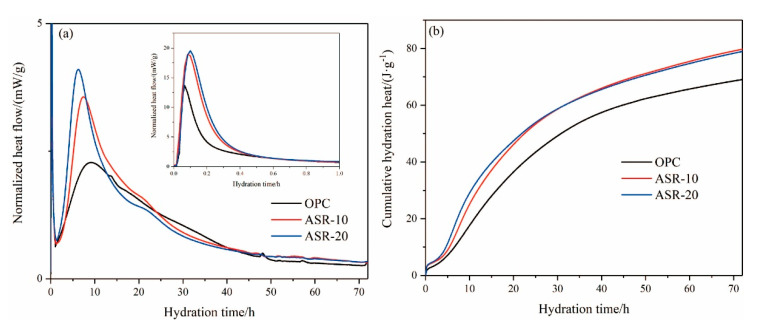
The hydration heat of varying pastes within 72 h: (**a**) normalized heat flow; (**b**) cumulative hydration heat.

The hydration heat of cementitious materials can reflect the hydration rate and has an important influence on the setting time and early strength of the slurry. Figure 6 shows the hydration heat release curves of ASR-cement composite cementitious materials over 72 h. Generally, the hydration heat release of cementitious materials can be divided into five stages [20]: (I) the rapid exothermic reaction, (II) the dormant period, (III) the acceleration, (IV) the deceleration, and (V) the steady state.

It can be seen from Figure 6a that the cementitious material reacted rapidly with water and released heat, indicating the rapid exothermic reaction stage (I). The released heat was mainly caused by the dissolution of raw material minerals in the liquid phase. After the addition of ASR, the exothermic heat of the rapid exothermic reaction stage increased significantly, indicating that the addition of ASR accelerated the dissociation of raw material particles. The dissociation rate increased at higher amounts of ASR. With the continuous hydration reaction, the raw materials released more heat after the dormant period (II) and entered the acceleration period (III). The addition of ASR caused the fast exothermic peak to become narrow and the maximum exothermic point to advance. The maximum exothermic peaks of OPC, ASR-10, and ASR-20 appeared at 9.18 h, 7.88 h, and 6.16 h, respectively, which indicates that ASR significantly promoted the hydration of cementitious materials and accelerated the hydration reaction.

In Figure 6b, the cumulative hydration heat release of the three groups of cementitious materials over 24 h followed the order ASR-20 > ASR-10 > OPC. This indicates that the addition of ASR increased the hydration heat release of cementitious materials in the early stages of hydration, and a higher ASR content increased the hydration heat release; however, after 72 h of hydration, the order of the cumulative hydration heat release became ASR-10 > ASR-20 > OPC, whose values were 78.89 J·g^−1^, 79.11 J·g^−1^, and 69.23 J·g^−1^, respectively. The main reason for this phenomenon was that the ASR does not have pozzolanic activity, so the main body of the hydration reaction is cement; thus, the later hydration reaction will lose its material support when the amount of cement is reduced. 

### 3.5. Pore Structure

The porosity and pore distribution of the hardened pastes of cementitious materials can be used to study the hydration reaction of cement-based materials. In this respect, the cumulative pore volume reflects the overall compactness of a paste, which is positively related to the compressive strength of the paste, and the different pore distributions will affect the durability of pastes by affecting their shrinkage and impermeability [21]. The quantity, shape, and size of hydration products have important influences on the porosity and pore distribution of hardened pastes.

**Figure 7:**
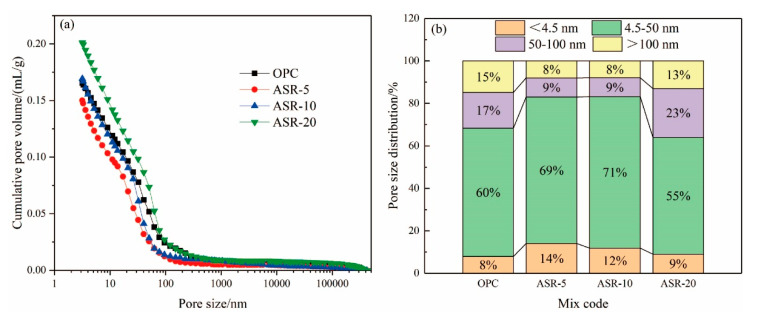
Pore structures of the ASR-cement hardened pastes after 3 days. Cumulative pore volume (**a**); pore size distributions (**b**).

From the cumulative pore volume of the hardened pastes with different ASR contents at 3 days in Figure 7a, the order of the cumulative pore volume from small to large is ASR-5, OPC, ASR-10, and ASR-20. This means that when 5% ASR was added, the cumulative pore volume of the hardened paste decreased. When the ASR content exceeded 10%, the cumulative pore volume of the hardened paste was greater than that of the blank control group (OPC), and the greater the ASR content, the greater the pore volume; therefore, from the point of view of cumulative pore volume, the ASR content in cementitious materials should not exceed 10%.

Figure 7b shows the pore distribution of the hardened pastes at 3 days. Many studies have shown that the pore distribution of pastes can be divided into four size ranges [22,23]: gel micropores (<4.5 nm), mesopores (4.5–50 nm), middle capillary pores (50–100 nm), and large capillary pores (>100 nm). A pore size larger than 50 nm can also be called harmful pores [24]. It can be seen from Figure 7b that when the alkali slag content is ≤ 10%, the volume of gel micropores and mesopores in the paste increased significantly, while the volume of middle capillary pores and large capillary pores decreased. In this respect, an appropriate amount of ASR can be added to reduce the proportion of harmful pores (>50 nm), which can improve the penetration resistance of the paste and optimize its durability.

There are two main effects of ASR on the pore structure of the pastes. One is the formation of a new hydration product, FS, with a 1–2 μm diameter. The second is that ASR is formed by the pressure filtration of very fine particles (<1 µm particle size), which play a physical filling role, thereby reducing the pore volume of the paste; however, the addition of ASR will reduce the amount of cement, thereby reducing the C-S-H gel and CH contents in the hydration products, especially when the ASR content exceeds 10%. The TG analysis also supports this. The pore structure of the paste is the result of the accumulation of C-S-H gels, FS, and CH, which explains why ASR-5 displayed the smallest cumulative pore volume and the best pore distribution.

### 3.6. Setting Time and Compressive Strength

**Figure 8:**
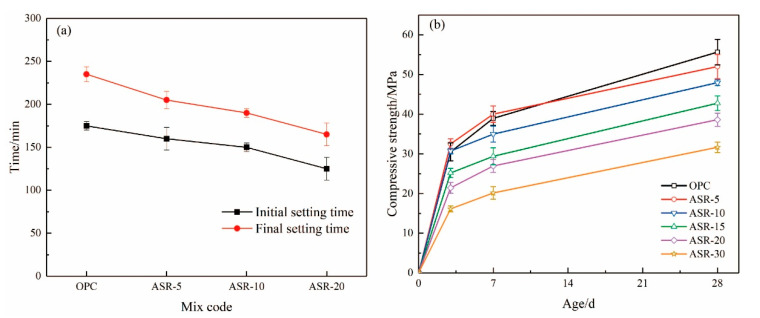
The setting time (**a**) of ASR-cement composite cementitious material and (**b**) mortar compressive strength.

Figure 8 shows the setting time of ASR-cement composite cementitious materials and the compressive strength of the resulting mortars. According to Figure 8a, the addition of ASR significantly shortened the setting time of the cementitious materials—the higher the ASR content, the shorter the initial setting time and final setting time. This is mainly because the addition of ASR accelerated the hydration rate of the cement, which is supported by the normalized heat flow of the cementitious materials (Figure 6a). Compared with the OPC control group, the final setting time of cementitious materials with 5%, 10%, and 20% ASR decreased by 30 min, 45 min, and 70 min, respectively. This was mainly because the addition of ASR accelerated the hydration rate of cement, which is also supported by the hydration heat results (Figure 6b).

The compressive strengths of the mortars test at 3 d, 7 d, and 28 d are shown in Figure 8b. At the 3 days age, the mortar sample with 5% ASR content had the highest compressive strength of 32.5 MPa, which is 6.6% higher than the OPC control group. The compressive strength of the mortar sample with an ASR content of 10% was basically the same as that of the OPC group. When it exceeded 10%, it was lower than the blank group OPC. The results mean that the addition of ASR improved the 3-day compressive strength of the mortar when its content did not exceed 10%. After curing for 7 days, only the ASR-5 group had a higher compressive strength than OPC, which was 40.02 MPa, an increase of 7.09%. The 28-day compressive strength of all samples containing ASR was lower than that of the OPC control group, indicating that the addition of ASR did not improve the long-term compressive strength of the mortar. A higher ASR content had a more negative effect on the long-term compressive strength.

## 4. Further Discussion

### 4.1. Hydration Mechanism

The mineral phase composition of ASR indicates that it has no pozzolanic activity; therefore, the hydration mechanism of ASR-cement composite cementing material mainly involves the activation of cement by ASR. According to the XRD and SEM analysis, the hydration products of ASR-cement composite cementitious materials were mainly C-S-H gel, CH, and FS. Among them, the C-S-H gel and CH formed mainly due to the reaction of silicate in cement and water (as shown in Equation (3)) [25], while the formation of FS was mainly caused by the reaction of aluminate in cement and chloride in ASR (as shown in Equation (4)) [26] or the reaction of sulfoaluminate and chloride salt in the liquid phase (as shown in Equation (5)) [27].

(3) $${3\mathrm{CaO}\cdot \mathrm{SiO}}_{2}{+\mathrm{nH}}_{2}O\to {\mathrm{xCaO}\cdot \mathrm{SiO}}_{2}{\cdot \mathrm{yH}}_{2}{O+(3-x)\mathrm{Ca}(\mathrm{OH})}_{2}$$

(4) $${3\mathrm{CaO}\cdot \mathrm{Al}}_{2}O_{3}{+2\mathrm{Ca}(\mathrm{OH})}_{2}{+2\mathrm{NaCl}+10H}_{2}O\to {3\mathrm{CaO}\cdot \mathrm{Al}}_{2}O_{3}{\cdot \mathrm{CaCl}}_{2}{\cdot 10H}_{2}O+2\mathrm{NaOH}$$

(5) $$\mathrm{AFm}+2\mathrm{NaCl}\;\to {\;3\mathrm{CaO}\cdot \mathrm{Al}}_{2}O_{3}{\cdot \mathrm{CaCl}}_{2}{\cdot 10H}_{2}{O+2\mathrm{NaSO}}_{4}{+2H}_{2}O$$

Equation (5) indicates that a small amount of gypsum in cement reacts with aluminate to form monosulfate (AFm, 3CaO·Al_2_O_3_·CaSO_4_). In the presence of Cl^−^ in a liquid environment, the Cl^-^ will replace SO_4_^2-^ in AFm to generate FS [28].

Figure 5 shows that the addition of ASR reduces the theoretical content of CH in the paste, indicating that part of the CH is consumed during hydration. This possibly occurred via its consumption during FS formation (Equation (4)). Another chloride-containing hydration product, calcium oxychloride (3CaO·CaCl_2_·15H_2_O), is also likely to exist in the paste (Equation (6)) [29]. The formation of calcium oxychloride also consumes CH, but its formation conditions generally require a higher CaCl_2_ concentration. There is also a competitive relationship between the formation of calcium oxychloride and the formation of FS, so the amount of calcium oxychloride is lower, so it was not observed in the characterization results, but many studies have proved its existence [30,31].

(6) $${12H}_{2}{O+\mathrm{Ca}}^{2+}{+2\mathrm{Cl}}^{-}+3\mathrm{Ca}{\left(\mathrm{OH}\right)}_{2}\to {3\mathrm{CaO}\cdot \mathrm{Ca}\mathrm{Cl}}_{2}{\cdot 15H}_{2}O$$

According to the hydration heat and TG-DSC results, ASR has an obvious effect on the early activation of cement, which is mainly dominated by chloride salts and CaCO_3_. The activation effect of chloride salts on cement is mainly reflected in two aspects: firstly, it can accelerate the dissociation rate of silicates (C_3_S and C_2_S), thus accelerating the hydration reaction rate. Secondly, the formation of FS consumes the aluminate ions in the liquid phase and, therefore, accelerates the dissociation rate of aluminate in the liquid phase. The activation effect of CaCO_3_ on cement is also mainly reflected in two aspects. One is that CaCO_3_ can accelerate the hydration rate of silicate, and the other is that CaCO_3_ particles can serve as nucleation sites in the liquid phase. The hydration products can quickly adhere to its surface after forming, so that the hydration reaction can continue in the liquid phase. In this respect, a faster hydration rate means a higher hydration heat release and shorter setting time, which is consistent with the results in Figure 4 and Figure 8a.

### 4.2. Applications

The characteristics of ASR-cement composite cementitious materials prevent their use in many fields. First, they cannot be used to prepare reinforced concrete because the chloride salts in ASR can corrode steel [32]. Secondly, they cannot be used to prepare mass concrete because the hydration heat is higher than that of OPC, which increases the concrete temperature and the cracking risk. Finally, ASR contains carbonates, which decrease the sulfate resistance of concrete [33].

The results of this study shared some similarities and differences with those of other studies [1,9,10,11,12]. The common point is that ASR shortened the setting time of cementitious materials, whether it is used with cement or with GGBS or FA materials. There are two main differences. One is that when ASR is used together with cement, it improves the pore structure of the paste because the volume of pores >50 nm was reduced, but this result is not clear in other studies. Secondly, when ASR was used with cement, its 28-day compressive strength was poor. Li [34] obtained the same result when using ASR to burn magnesium oxychloride cement, but, when used with GGBS-based materials, its 3- and 28-day compressive strength was significantly improved.

Overall, the applications of ASR-cement composite cementitious materials have several potential development directions. One is the preparation of special-purpose concrete, such as for prefabricated components, because they can greatly improve the production efficiency. They can also be used to produce road concrete, slope stones, and artificial reef concrete, because soda plants are located near the sea [35]. Second, they can be used in general building materials, such as building mortar and bricks. Third, they can be used as a binder for mine filling materials. Finally, the costs of using ASR are almost negligible, which gives ASR-cement composite cementing materials good economic benefits—the higher the amount of ASR used, the better the economic benefits.

In addition, the application scope of ASR-cement composite cementitious materials can be broadened if it is used together with SCMs with high aluminate content (e.g., GGBS and FA). Some researchers [35,36] have studied the properties of ASR-GGBS materials, and the results showed that ASR activates GGBS; however, it also has significant disadvantages, such as low compressive strength in the early stage (3 days) and a high proportion of harmful pore sizes in the hardened paste. In Figure 7 and Figure 8, when ASR and cement are used together, volume of the harmful pore is reduced when using 10% ASR, and the 3-day compressive strength was also significantly increased. It seems that a more optimal paste pore structure can be obtained if the ASR-cement cementitious material is used with GGBS. In this respect, using ASR-cement composite cementitious materials and SCMs with a high aluminate content can be used to prepare cementitious materials with better performance, but this requires further research.

## 5. Conclusions

In this paper, a composite cementing material was prepared using ASR and cement, and its hydration mechanism and hydration characteristics were studied using XRD, SEM, TG-DSC, and MIP. The main conclusions are as follows:

The main hydration products of ASR-cement were C-S-H gel, CH, and FS. The shape of FS was hexagonal plate-like CH but smaller in size (1–2 μm). The amount of FS produced depends on the ASR content in the cementitious material, and more FS was produced at a higher ASR dosage. Meanwhile, the addition of ASR also reduced the theoretical content of CH in the paste, mainly because CH was consumed during the hydration reaction. 

The addition of ASR improved the pore structure of ASR-cement paste by decreasing the number of harmful pores (>50 nm). The paste displayed the optimal pore structure at an ASR content of 5%. Meanwhile, ASR increased the heat of hydration of cementitious materials, mainly because chlorides and carbonates accelerated the hydration of silicates and aluminates. Correspondingly, the setting time of the cementitious material also decreased upon increasing the ASR content.

The early compressive strength of the mortar was significantly improved when the ASR content in the cementitious material exceeded 10%, but the long-term compressive strength was poor regardless of the ASR content; therefore, there are disadvantages in the mechanical properties of ASR-cement cementitious materials, but they have advantages in their working performance.

ASR-cement composite cementing materials can be applied in plain concrete without steel bars and are especially suitable for preparing marine concrete, road concrete, etc. When used with GGBS or fly ash, they will have better performance and can also broaden their applications. Overall, the ASR-cement composite cementitious materials have significant advantages in terms of costs and reducing CO_2_ emissions of the cement industry.

## Figures and Tables

**Table 1:** Chemical composition of raw materials (mass fraction/%).

Composition	Cement	ASR
CaO	62.38	52.88
SiO_2_	22.11	10.19
MgO	2.28	8.35
SO_3_	2.62	8.87
Na_2_O	1.73	1.84
Al_2_O_3_	4.43	3.25
Fe_2_O_3_	3.13	1.23
K_2_O	0.26	0.38
TiO_2_	0.35	0.19
MnO	0.3	0.06
Cl	0.012	11.95

**Table 2:** Mix proportions of the pastes with different amounts of ASR (g).

Mix Code	Cement	ASR	Water
OPC	100	0	50
ASR-5	95	5	50
ASR-10	90	10	50
ASR-20	80	20	50
ASR-30	70	30	50

## Data Availability

The data presented in this study are available on request from the corresponding author. The data are not publicly available due to the privacy restrictions.
